# Diagnosis of Atrial Fibrillation Using Machine Learning With Wearable Devices After Cardiac Surgery: Algorithm Development Study

**DOI:** 10.2196/35396

**Published:** 2022-08-01

**Authors:** Daisuke Hiraoka, Tomohiko Inui, Eiryo Kawakami, Megumi Oya, Ayumu Tsuji, Koya Honma, Yohei Kawasaki, Yoshihito Ozawa, Yuki Shiko, Hideki Ueda, Hiroki Kohno, Kaoru Matsuura, Michiko Watanabe, Yasunori Yakita, Goro Matsumiya

**Affiliations:** 1 Department of Cardiovascular Surgery University of Chiba Chiba Japan; 2 Department of Artificial Intelligence Medicine Graduate School of Medicine University of Chiba Chiba Japan; 3 RIKEN Information R&D and Strategy Headquarters Yokohama Japan; 4 Clinical Research Center University of Chiba Chiba Japan

**Keywords:** wearable device, atrial fibrillation, photoplethysmography, cardiology, heart, mHealth, mobile health, pulse, development, pilot study, Apple Watch, sensor, algorithm, detection, diagnose, cardiac surgery, machine learning

## Abstract

**Background:**

Some attempts have been made to detect atrial fibrillation (AF) with a wearable device equipped with photoelectric volumetric pulse wave technology, and it is expected to be applied under real clinical conditions.

**Objective:**

This study is the second part of a 2-phase study aimed at developing a method for immediate detection of paroxysmal AF, using a wearable device with built-in photoplethysmography (PPG). The objective of this study is to develop an algorithm to immediately diagnose AF by an Apple Watch equipped with a PPG sensor that is worn by patients undergoing cardiac surgery and to use machine learning on the pulse data output from the device.

**Methods:**

A total of 80 patients who underwent cardiac surgery at a single institution between June 2020 and March 2021 were monitored for postoperative AF, using a telemetry-monitored electrocardiogram (ECG) and an Apple Watch. AF was diagnosed by qualified physicians from telemetry-monitored ECGs and 12-lead ECGs; a diagnostic algorithm was developed using machine learning on the pulse rate data output from the Apple Watch.

**Results:**

One of the 80 patients was excluded from the analysis due to redness caused by wearing the Apple Watch. Of 79 patients, 27 (34.2%) developed AF, and 199 events of AF including brief AF were observed. Of them, 18 events of AF lasting longer than 1 hour were observed, and cross-correlation analysis showed that pulse rate measured by Apple Watch was strongly correlated (cross-correlation functions [CCF]: 0.6-0.8) with 8 events and very strongly correlated (CCF>0.8) with 3 events. The diagnostic accuracy by machine learning was 0.9416 (sensitivity 0.909 and specificity 0.838 at the point closest to the top left) for the area under the receiver operating characteristic curve.

**Conclusions:**

We were able to safely monitor pulse rate in patients who wore an Apple Watch after cardiac surgery. Although the pulse rate measured by the PPG sensor does not follow the heart rate recorded by telemetry-monitored ECGs in some parts, which may reduce the accuracy of AF diagnosis by machine learning, we have shown the possibility of clinical application of using only the pulse rate collected by the PPG sensor for the early detection of AF.

## Introduction

Atrial fibrillation (AF) is the most common, persistent arrhythmia in adults, with a lifetime risk of 25%-33%, and it is associated with heart failure, stroke, dementia, and death [1,2]. However, approximately one-third of patients with paroxysmal AF are asymptomatic, making it difficult to diagnose an arrhythmia without symptoms unless an ECG monitoring is performed in a medical facility [1,3-5]. In fact, up to 50% of patients who have a stroke due to AF are diagnosed with AF after the stroke has occurred [6-9].

The European Society of Cardiology guidelines recommends opportunistic screening for AF using pulse checks or ECG rhythm strips in patients over 65 years of age [10]. With the recent advent of mobile devices and wearable sensors, it has become possible to continuously monitor health status in daily life. The number of connected wearable devices worldwide has more than doubled in 3 years, from 325 million in 2016 to 722 million in 2019; by 2022, the number of devices is expected to reach more than 1 billion. In addition, their sales are expected to grow from US $14.93 billion in 2020 to US $17.35 billion in 2021, at a compound annual growth rate of 16.2%, making wearable devices increasingly popular [11,12].

Recent smartwatches are equipped with photoplethysmography (PPG) that uses infrared light-emitting diode optical sensors to monitor changes in microvascular blood volume, making it possible to continuously monitor pulse rate in daily life. The clinical applicability of PPG has been addressed in several studies, one of the most famous is the Apple Heart Study [13]. Of the participants who received arrhythmia notifications from their Apple Watch, more than one-third had AF confirmed by a subsequently worn ECG patch monitoring, and of the arrhythmia notifiers who returned their ECG patches, 84% (72/86) had a positive ECG patch (ie, positive for AF). Positive notifications were consistent with AF 84% of the time (95% CI 76-92) [13].

We previously measured pulse rate in patients after cardiac surgery wearing 2 wearable devices and showed that heart rate in sinus rhythm correlated well with pulse rate measured by wearable devices; however, during AF, the accuracy was slightly lower, but the pulse rate tracking was accurate enough for clinical applications [14].

Further innovations in wearable devices have led to the technology of single-lead ECG collection. By touching the wearable device with the contralateral finger for about 30 seconds, a single-lead ECG can be taken, and the ECG data can be used to diagnose AF. Although this method is by a single lead, it has been shown to have a higher sensitivity and specificity due to the ECG information such as P waves and QRS waves [9,15-17].

However, many patients with AF are older people, and it is difficult to have them voluntarily perform the task of touching the wearable device with the contralateral finger for 30 seconds to collect a single-lead ECG when they are notified of an arrhythmia.

There have been many attempts to detect arrhythmias using wearable devices, but most of these studies have been based on brief observations and data collection under ideal conditions in selected patients. In this study, we conducted long-term pulse rate monitoring during hospitalization of patients undergoing cardiac surgery to collect data under real clinical conditions.

This is the second part of a 2-phase study aimed at developing a method for the immediate detection and diagnosis of paroxysmal AF using a wearable device with a PPG sensor. We investigated the possibility of clinical application of heart rate monitoring by wearable devices in the special environment post cardiac surgery.

## Methods

### Ethics Approval

In accordance with all applicable regulations, this study was approved by the Clinical Research Ethics Committee of Chiba University Hospital (Clinical Research Protocol jRCTs032200032) on October 23, 2020. We obtained written informed consent from all study participants to allow data monitoring as well as data registration and management to be performed by the Chiba University Hospital Clinical Trials Data Center. In addition, an independent Data Monitoring Committee has been established within the Department of Clinical Trials at Chiba University.

### Participants

Between June 2020 and March 2021, 80 patients scheduled for cardiovascular surgery at a single institution were recruited for this study. The exclusion criteria were the following: a history of permanent pacemaker implantation, skin disorders at the wristband site, and past hypersensitivity to wristbands (or rubber products); in addition, patients with chronic AF who did not undergo arrhythmia surgery and patients who were unfit for the safe conduct of this study according to the principal investigator or subinvestigator were also excluded.

After written informed consent was obtained, 80 subjects were given an Apple Watch (series 4) and asked to wear it on one forearm. A spare, fully charged smartwatch was always available to measure their pulse for 24 hours continuously. The Apple Watch was removed on the day the patient underwent surgery; the Apple Watch was reapplied when the patient was discharged from the intensive care unit (ICU) to the general ward after cardiac surgery (ie, usually the day after the surgery); all Apple Watch wearing was done under the supervision of a physician. Apple Watch was worn continuously until discharge or for up to 14 days after discharge from the ICU unless the study was interrupted for clinical or personal reasons.

A publicly available HeartWatch mobile app (Tantsissa) was used to access data from a standard commercially available Apple Watch.

Apple Watch has 2 functional modes for measuring pulse rate: standby mode and workout mode. In standby mode, the pulse rate is measured once every few minutes, resulting in less data. Therefore, all participants’ Apple Watches were set to workout mode during wear.

Central ECG monitoring using a telemetry system (VitalSignTelemater GZ-130P; Nihon Kohden) was continued in all patients until hospital discharge. If AF was suspected, a 12-lead ECG monitoring was performed, whenever possible, for confirmation.

AF was diagnosed based on the guided diagnostic criteria by 2 qualified physicians [10].

When an arrhythmia was identified, the telemetry data were checked, and the time of the onset and cessation of arrhythmia was recorded. These procedures were repeated each time an arrhythmia was suspected on the central monitor.

### Heart Rate and Pulse Rate Measurements

Heart rate data were obtained from a telemetry electrocardiograph that calculates the heart rate every 3 seconds based on the previous RR interval.

Pulse rate data were obtained from an Apple Watch with built-in PPG; in Apple Watch workout mode, the pulse rate is calculated every 5-6 seconds. The time interval of each pulse rate calculation on the Apple Watch may vary depending on the conditions (eg, low perfusion conditions and dark skin color) because the Apple Watch has an automatic optimization function that increases the brightness of the light-emitting diodes and the sampling rate to compensate for low signal levels [18].

### Cross-Correlation Analysis

Using the same technique as in part 1 of this study [14], we created time series pulse rate trends and heart rate trends and analyzed the similarity of the trend patterns.

Events in patients with brief AF or bouncing between AF and atrial flutter have negative impacts on correlation analysis; therefore, cross-correlation analysis was performed on AF events lasting longer than 60 minutes that transitioned from sinus rhythm to AF and from AF back to sinus rhythm, as measured by central ECG monitoring and the Apple Watch. The complete pulse rate data measured by the central ECG and Apple Watch were included in the analysis.

We then used the trend data to check the accuracy of PPG-based pulse rate measurement with reference to ECG-based heart rate measurement during AF.

### Machine Learning Model for AF Diagnosis

We used a gradient boosting decision tree (GBDT) [19] to diagnose AF based on pulse rate data from the Apple Watch and patient background information summarized in Table S1 in Multimedia Appendix 1. We computed features of the pulse rate data every minute and treated them as a single record for training and validation of the GBDT model. As pulse rate features, the mean and SD of pulse rate per minute within 10 minutes of the timing for the AF diagnosis were calculated. The median value of the mean and SD of heart rate up to the time of diagnosis was used as the baseline. GBDT is a kind of ensemble learning that can handle missing values without complementation and is known to provide high prediction accuracy by boosting technology. The Python package LightGBM (version 3.3.2; Python Software foundation) was used for the GBDT implementation, and hyperparameter was tuned by Bayesian optimization with cross-validation, using the Optuna framework [20]. The data of 79 patients were first split into a training cohort of 59 patients and a test cohort of 20 patients; only the training cohort was used for hyperparameter tuning, variable selection, and training of the machine learning model. The variable importance of the GBDT model was calculated for the training cohort by the “permutation importance” function implemented in the Python package eli5 (version 0.11.0; Python Software foundation). To evaluate the performance of the machine learning model for AF diagnosis, a receiver operating characteristic (ROC) analysis was performed. The ROC analysis plots the change in sensitivity and specificity, as the threshold for the probability of AF is varied. The ROC analysis was performed using the R package pROC (version 1.18.0; Xavier Robin).

### Other Statistical Analysis

Summary of statistics are presented as frequency and percentage for categorical data, mean (SD) for continuous variables, and frequency and percentage for categorical variables.

All statistical analyses were performed using SAS (version 9.4 for Windows; SAS Institute Inc).

## Results

### Patient Background

Of the 80 patients, 1 had redness on the area the Apple Watch was worn, and we stopped the study before she underwent surgery. The demographics of the 79 patients are shown in Table 1.

**Table 1:** Characteristics of study participants (N=79).

Demographics	Values
Age (years), mean (SD)	65.8 (13.4)
Sex (male), n (%)	57 (72.2)
Left ventricular ejection fraction, mean (SD)	58.9 (8.8)
Off-pump coronary artery bypass grafting, n (%)	18 (21.5)
Valve surgery, n (%)^a^	57 (72.2)
Other surgery, n (%)^b^	4 (5.1)
Minimally invasive cardiac surgery, n (%)	7 (8.9)
Monitoring period (days), mean (SD)	13.3 (2.5)
Use of antiarrhythmic drugs before the event, n (%)^c^	30 (38.0)

^a^Included multiple surgeries.

^b^Included 2 thoracic surgeries, 1 atrial septal defect closure, and 1 ventricular septal myectomy, and on-pump coronary-artery bypass grafting.

^c^Types of antiarrhythmic drugs included pilsicainide, flecainide, amiodarone, verapamil, digoxin cibenzoline, sotalol, bepridil, and β-blockers.

### Arrhythmias

The mean number of days of smartwatch wear per patient was 13.3 (SD 2.5) days, and the median number of days of wear was 14 (IQR 11-15) days. Of 79 patients, 69 had a good postoperative course and were discharged within 14 days after transfer from ICUs to the general ward; 10 patients required more time for treatment of complications or postoperative rehabilitation, so pulse rate measurement with the Apple Watch was ended 14 days after leaving the ICU as per protocol.

The total number of arrhythmia events diagnosed in the 79 patients included in the analysis was 429 in 31 patients. The total number of AF events was 199 in 27 patients (Table 2).

**Table 2:** Arrhythmia events in patients (N=79).

Event	Patients, n (%)	Events, n	Total time (h)
Atrial fibrillation	27 (34.2)	199	713.7
Atrial fibrillation and atrial flutter^a^	1 (1.3)	13	4.2
Atrial flutter	7 (8.9)	37	163.8
Atrial flutter and premature atrial contractions^b^	1 (1.3)	3	5.4
Atrial tachycardia	6 (7.6)	97	114.9
Paroxysmal supraventricular tachycardia	1 (1.3)	10	0.8
Ventricular tachycardia	1 (1.3)	67	0.6
Other^c^	1 (1.3)	3	61.7

^a^Atrial fibrillation and atrial flutter are mixed.

^b^Atrial flutter and premature atrial contractions are mixed.

^c^Included sinus arrest and unidentified arrhythmias.

### Cross-Correlation Analysis of Pulse Rate Accuracy Based on PPG Sensor During AF

Table 3 shows the results of the CCF analysis of the 1-minute moving average of the central monitor ECG heart rate and Apple Watch pulse rate in the 18 AF events lasting more than 1 hour used in the analysis.

Of the 18 events, 8 events had a strong correlation (CCF: 0.6-0.8), and 3 events had a very strong correlation (CCF>0.8) [21].

**Figure 1:**
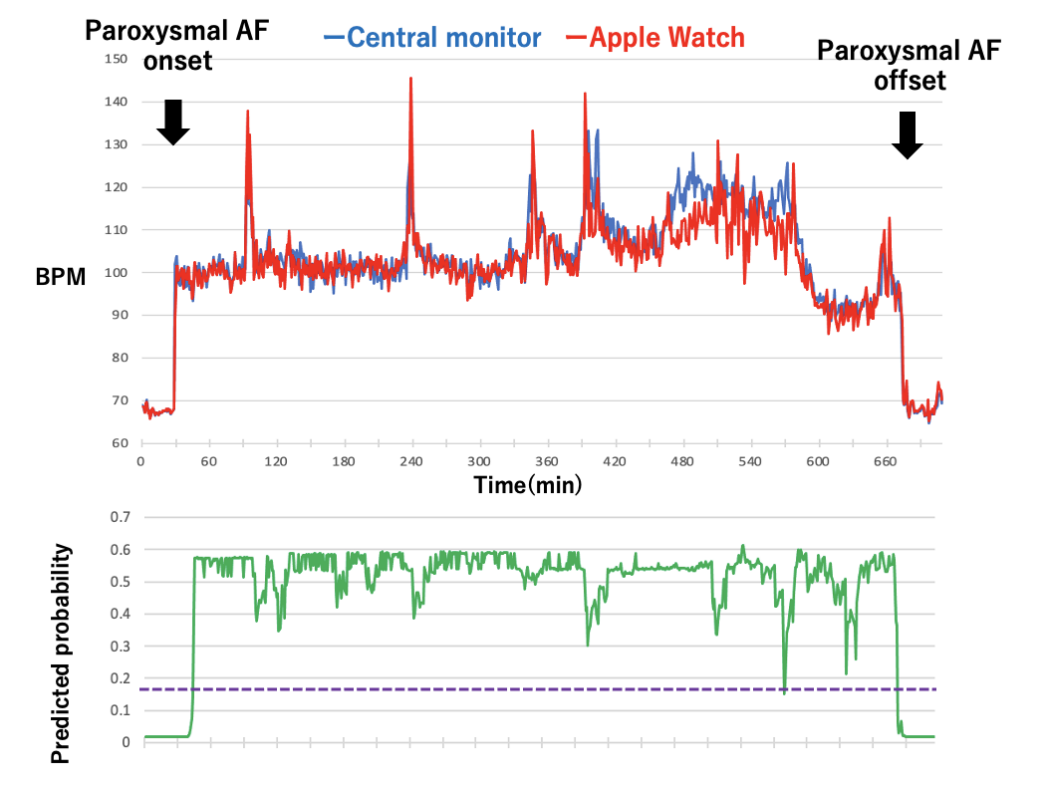
Time series trend curves during atrial fibrillation (AF) (event 12). The figure compares the trend curve of the Apple Watch (red curve) with central monitor heart rate (blue curve). The green curve shows the time trend of AF diagnosis prediction rate by machine learning. The purple dotted line indicates the diagnostic threshold for AF (0.018). BPM: beats per minute.

**Figure 2:**
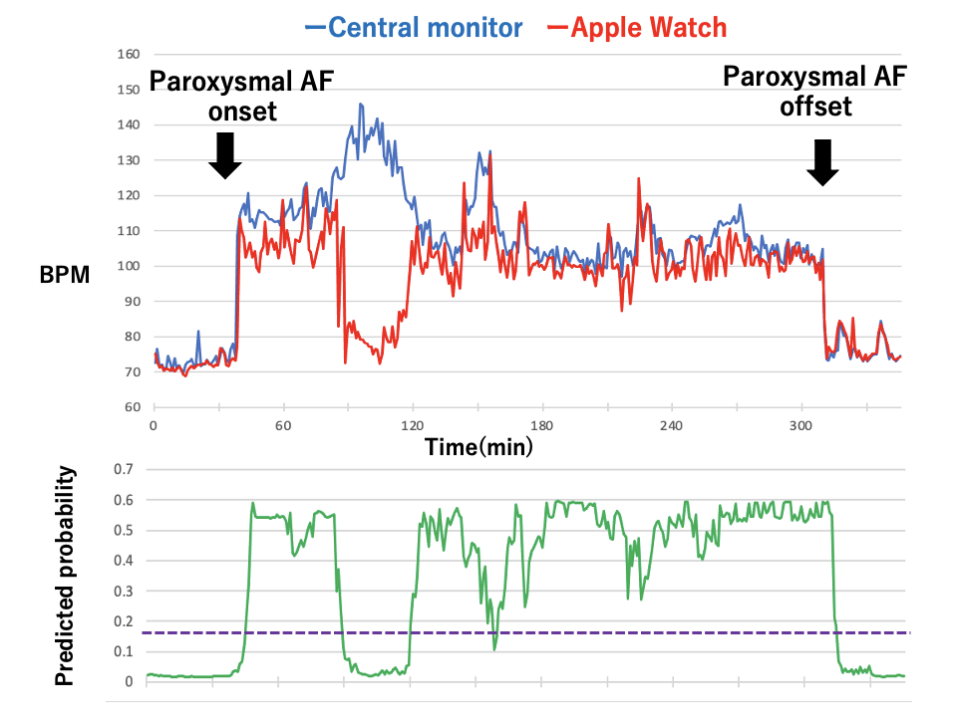
Time series trend curves during atrial fibrillation (AF) for event 11. The figure compares the trend curve of the Apple Watch (red curve) with central monitor heart rate (blue curve). The green curve shows the time trend of AF diagnosis prediction rate by machine learning. The purple dotted line indicates the diagnostic threshold for AF (0.018). BPM: beats per minute.

Figure 1 shows 2 time series curves (one for heart rate trend and the other for pulse rate trend) for one event that showed a very strong correlation (Event 12), and Figure 2 presents another event that showed a weak correlation (Event 11). In Figure 1, the Apple Watch pulse rate follows the central monitor ECG heart rate very well, but in Figure 2, the Apple Watch pulse rate does not follow the central monitor ECG heart rate after the heart rate exceeds about 120 per minute and is measured as a low value.

**Table 3:** Time series correlation of pulse change in paroxysmal atrial fibrillation.

Event number	Cross-correlation function^a^	*P* value
1	.69869	<.001
2	.06296	.33
3	.17394	.001
4	.35918	<.001
5	.81694	<.001
6	.76119	<.001
7	–.19029	.07
8	.64214	<.001
9	.77215	<.001
10	.76217	<.001
11	.02752	.64
12	.88082	<.001
13	.60062	<.001
14	.84744	<.001
15	.64360	<.001
16	.41605	<.001
17	.17998	.14
18	.75319	<.001

^a^For reference, the strength of correlation [20] can be classified in the literature as follows: <0.19 as very weak; 0.2-0.39 as weak; 0.4-0.59 as moderate; 0.6-0.79 as strong; and >0.8 as very strong.

### Diagnosis of AF by Machine Learning

**Figure 3:**
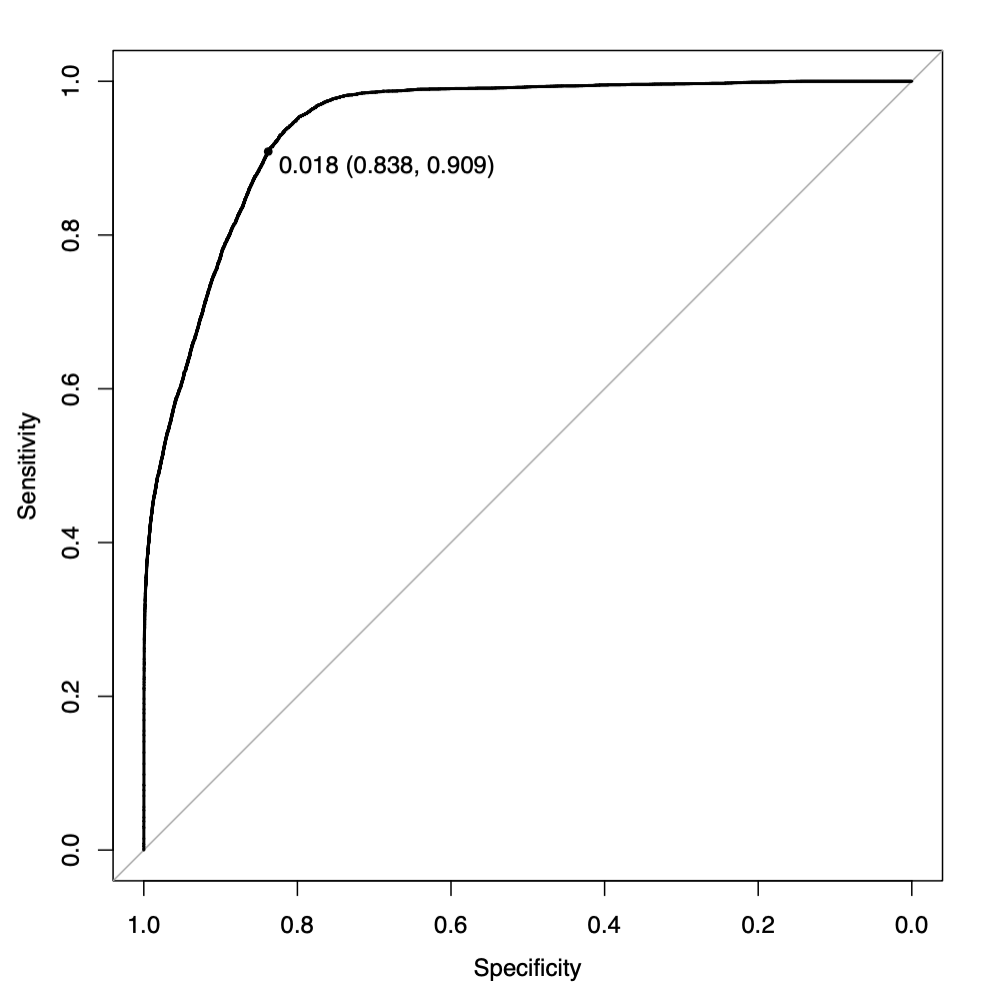
Receiver operating characteristic curve of atrial fibrillation diagnosis rate.

**Figure 4:**
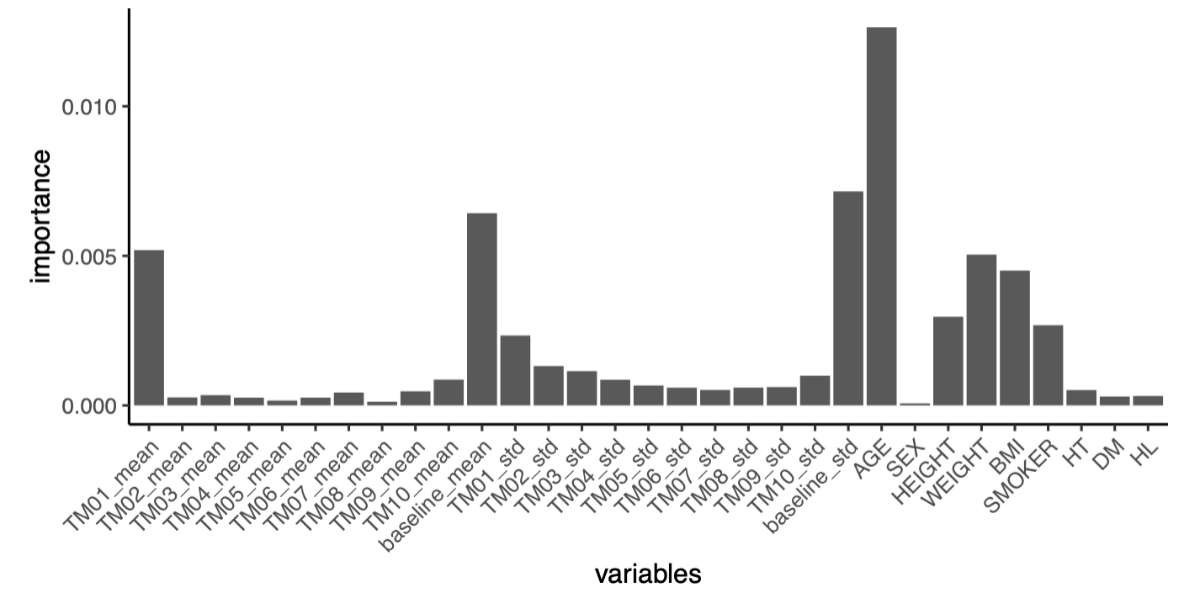
Factors contributing to the diagnosis of atrial fibrillation. DM: diabetes mellitus; HL: hyperlipidemia; HT: hypertension; TM01_mean: mean heart rate 1 minute before the timing; TM02_mean: mean heart rate 2 minutes before the timing; TM03_mean: mean heart rate 3 minutes before the timing; TM10_mean: mean heart rate 10 minutes before the timing; baseline_mean: mean heart rate at the timing; TM01_std: SD 1 minute before the timing; TM02_std; SD 2 minutes before the timing; TM03_std: SD 3 minutes before the timing; TM10_std: SD 10 minutes before the timing.

In this study, the area under the ROC curve for the diagnosis of AF with the GBDT model was 0.9416, with a sensitivity of 0.909 and a specificity of 0.838 at the point closest to the top left (Figure 3; Table 4). The importance of the GBDT model is shown in Figure 4. The importance of age and baseline SD was high. In Figure 1 and Figure 2, the diagnostic predictive rate of AF is plotted as time series, and in Figure 2, where the Apple Watch pulse rate did not track the actual heart rate, the diagnostic rate dropped notably at that site.

**Table 4:** The sensitivity and specificity of the gradient boosting decision tree (GBDT) atrial fibrillation (AF) prediction.

	Gold standard diagnosis		
	AF positive	AF negative	Total
GBDT AF prediction			
AF positive	8113	25,622	33,735
AF negative	816	132,515	133,331
Total	8929	158,137	167,066
Sensitivity (%)	90.9	—^a^	—
Specificity (%)	83.8	—	—

^a^Not applicable.

## Discussion

### Principal Findings

In this study, we measured the pulse rate of 79 patients undergoing cardiac surgery by a wearable device on their forearm for 24 hours during hospitalization and continuously until discharge (for a maximum of 14 days after discharge from the ICU). The pulse rate data obtained were used in a machine learning model to create a diagnostic algorithm for AF. The diagnostic accuracy of this algorithm was 0.9416 (sensitivity 0.909 and specificity 0.838 at the point closest to the top left) for the area under the ROC curve. Some authors have reported that the diagnostic accuracy of AF by wearable devices is high with an area under the ROC curve of 0.9 or higher [22-24]. However, these are based on data sampling in a limited ideal environment (eg, at rest, or when data collection time is only a few hours) and with a preselected group of patients with a history of AF. For patients after cardiac surgery who are prone to various arrhythmias other than AF, these studies have a large proof-of-concept aspect, and it is desirable to conduct research and development for practical use in the real world of patients with cardiac diseases.

In this study, we measured pulse rates under real clinical conditions and were able to diagnose AF with high accuracy, using only time series pulse rate data and patient background information. The Apple Watch with a silicon band was worn by patients, and although 1 patient complained of an itchy sensation with redness at the site of wearing, no other adverse events caused by the Apple Watch occurred. Some patients, after coronary artery bypass surgery, had radial artery grafts and forearm wounds extending to the wrist, which limited the use of the watch in some cases; however, the watch could be worn and monitored for a long time in clinical practice. In the perioperative period, various arrhythmias other than AF occur, as well as circulatory conditions such as water overflow and dehydration. To the best of our knowledge, no study has attempted continuous and prolonged monitoring and machine learning for AF diagnosis using a wearable device in such an environment.

In this study, the cross-correlation coefficient between the heart rate obtained from central ECG monitoring and the pulse rate data obtained from the Apple Watch for AF lasting more than 1 hour showed a strong correlation in 11 out of 18 events. As shown in Figure 2, there were cases where the Apple Watch pulse rate did not follow the timing of the faster heart rate. Al-Kaisey et al [25] also reported that pulse rate during AF is underestimated compared to sinus rhythm. Since the PPG sensor is a pulse pressure waveform that originates from heart contraction and propagates through the cardiovascular system, as shown in our previous study [14], depending on pathophysiological conditions such as intravascular volume, heart rate, and heart condition, sufficient pulse pressure may not be generated [14,26-28]. For the above reasons, pulse rate data obtained from PPG sensors may not accurately represent the heart rate, especially in patients with cardiac disease or after cardiac surgery, making it difficult to diagnose AF. In this study, as shown in Figure 2, the diagnostic accuracy dropped where the Apple Watch pulse rate did not track the actual heart rate.

The main factors contributing to the AF diagnosis with GBDT were age and baseline SD. There have been many attempts to use machine learning to diagnose AF; however, to our knowledge, there have not been studies that have identified the factors contributing to its diagnosis. Although the machine learning in this study was not able to achieve a high diagnostic accuracy that could be immediately applied in actual clinical practices, it suggests the possibility that a wearable device can be used to monitor pulse rate for a long period of time and diagnose AF in the special environment after cardiac surgery.

### Limitations

There are several limitations to this study, one of which is the small sample size. We believe that we sampled a relatively large number of patients in a study of long-term monitoring under real clinical conditions, but the sample size is still too small for machine learning. Second, the Apple Watch wearing process was done entirely by a physician, and when the patient removed the watch by himself, the heart rate measurement stopped, and some data were missing. The data collected from the Apple Watch were synchronized each time the Apple Watch was charged and were stored in the iPhone, but there were times when data were not transferred to the iPhone for some unknown reason, resulting in missing data.

### Clinical Outlook

In this study, we developed an algorithm to immediately diagnose AF, using machine learning on the collected data; however, there are reports indicating that analysis using deep learning as an artificial intelligence technology can diagnose AF with higher accuracy [29].

We are planning to create a classifier with higher accuracy and diagnostic performance by using a new diagnostic algorithm based on deep learning. Once a classifier with enough accuracy to withstand clinical use is completed, patients will be able to know their own arrhythmia in real time, and heart rhythms can be monitored safely with a very simple wristwatch device. It is said that potential AF is involved in the development of cerebral infarction and stroke, but there is little evidence on the threshold for developing cerebral infarction, which remains controversial [30]. If it becomes possible to accurately record AF at all stages, this is expected to become an extremely useful tool to determine the threshold for stroke onset and may lead to the introduction of anticoagulation therapy in appropriate patient groups to prevent stroke.

### Conclusions

In the second phase of the 2-phase study, the Apple Watch was safely worn for a long period of time even in the special environment post heart surgery. Furthermore, the findings of this study showed that the Apple Watch could potentially detect AF with a machine learning classifier during the recovery period after heart surgery. The next step will be to improve the accuracy of immediate diagnosis of AF by deep learning.

## Appendix

Multimedia Appendix 1 Patients' background information.

## Abbreviations

AF: atrial fibrillation
CCF: cross-correlation function
ECG: electrocardiogram
GBDT: gradient boosting decision tree
ICU: intensive care unit
PPG: photoplethysmography
ROC: receiver operating characteristic
