# Supplementary material for: Diagnosis of Atrial Fibrillation Using Machine Learning With Wearable Devices After Cardiac Surgery: Algorithm Development Study
Source: JMIR Form Res. 2022 Aug 1;6(8):e35396. doi: 10.2196/35396 (PMC9379796; doi:10.2196/35396)
Supplement: Multimedia Appendix 1 [file formative_v6i8e35396_app1.docx]

**Supplementary Table S1.** Statistics of background information of patients

| **Characteristic** | **train**, N = 59*^1^* | **test**, N = 20*^1^* | **p-value***^2^* |
| --- | --- | --- | --- |
| AGE | 70 (56, 76) | 72 (57, 76) | 0.5 |
| SEX=male | 42 (71%) | 15 (75%) | 0.7 |
| HEIGHT | 166 (159, 169) | 167 (160, 173) | 0.4 |
| WEIGHT | 64 (54, 71) | 65 (61, 71) | 0.4 |
| BMI | 23.2 (20.8, 25.6) | 24.6 (22.6, 25.6) | 0.3 |
| SMOKER |  |  | 0.5 |
| 0 (never) | 20 (34%) | 10 (50%) |  |
| 1 (ex) | 10 (17%) | 2 (10%) |  |
| 2 (current) | 29 (49%) | 8 (40%) |  |
| HT | 43 (73%) | 14 (70%) | 0.8 |
| DM | 17 (29%) | 6 (30%) | >0.9 |
| HL | 30 (51%) | 9 (45%) | 0.7 |
| *^1^* Median (IQR); n (%) | | | |
| *^2^* Wilcoxon rank sum test; Pearson's Chi-squared test; Fisher's exact test | | | |
